# Period-aggregated transformer for learning latent seasonalities in long-horizon financial time series

**DOI:** 10.1371/journal.pone.0308488

**Published:** 2024-08-08

**Authors:** Zhenyang Tang, Jinshui Huang, Denisa Rinprasertmeechai

**Affiliations:** Southwestern University of Finance and Economics, Chengdu, China; Universidad de Granada, SPAIN

## Abstract

Fluctuations in the financial market are influenced by various driving forces and numerous factors. Traditional financial research aims to identify the factors influencing stock prices, and existing works construct a common neural network learning framework that learns temporal dependency using a fixed time window of historical information, such as RNN and LSTM models. However, these models only consider the short-term and point-to-point relationships within stock series. The financial market is a complex and dynamic system with many unobservable temporal patterns. Therefore, we propose an adaptive period-aggregation model called the Latent Period-Aggregated Stock Transformer (LPAST). The model integrates a variational autoencoder (VAE) with a period-to-period attention mechanism for multistep prediction in the financial time series. Additionally, we introduce a self-correlation learning method and routing mechanism to handle complex multi-period aggregations and information distribution. Main contributions include proposing a novel period-aggregation representation scheme, introducing a new attention mechanism, and validating the model’s superiority in long-horizon prediction tasks. The LPAST model demonstrates its potential and effectiveness in financial market prediction, highlighting its relevance in financial research and predictive analytics.

## Introduction

Fluctuations in the financial market result from a variety of driving forces and can be influenced by numerous factors. In traditional finance, researchers have aimed to identify the factors influencing stock prices. A notable model in this context is the Fama–French model, which is used to elucidate stock price fluctuations through three primary factors [1]. The factor model simplifies the market features that influenced by firm characteristics, investment decisions, and many other factors. Pilot studies have attempted to quantitatively dissect the dynamics of the stock market [2–5], using machine learning methods to further uncover the latent features in stock movement prediction via nonlinear representations [6]. This task is especially crucial for informing investment decisions and market regulations and, so, is receiving increased attention from many researchers and investors.

The stock market is a complex system, where the effectiveness of predictive tasks heavily relies on data quality. Traditional financial research, assuming certain distributional properties of stock data, has identified historical information as being crucial for stock prediction. However, stock data contain noise [7], and machine learning can be applied in financial research to process noise information and extract intrinsic characteristics. Inspired by other fields such as natural language processing, in which interference information is reduced via sequence modeling, machine learning has attained success in stock prediction through the use of supervised learning to capture temporal dependencies and predict returns for the next day. These temporal-domain analyses provide an intuitive perspective for archiving the characteristics of stock prices as they change over time.

**Fig 1:**
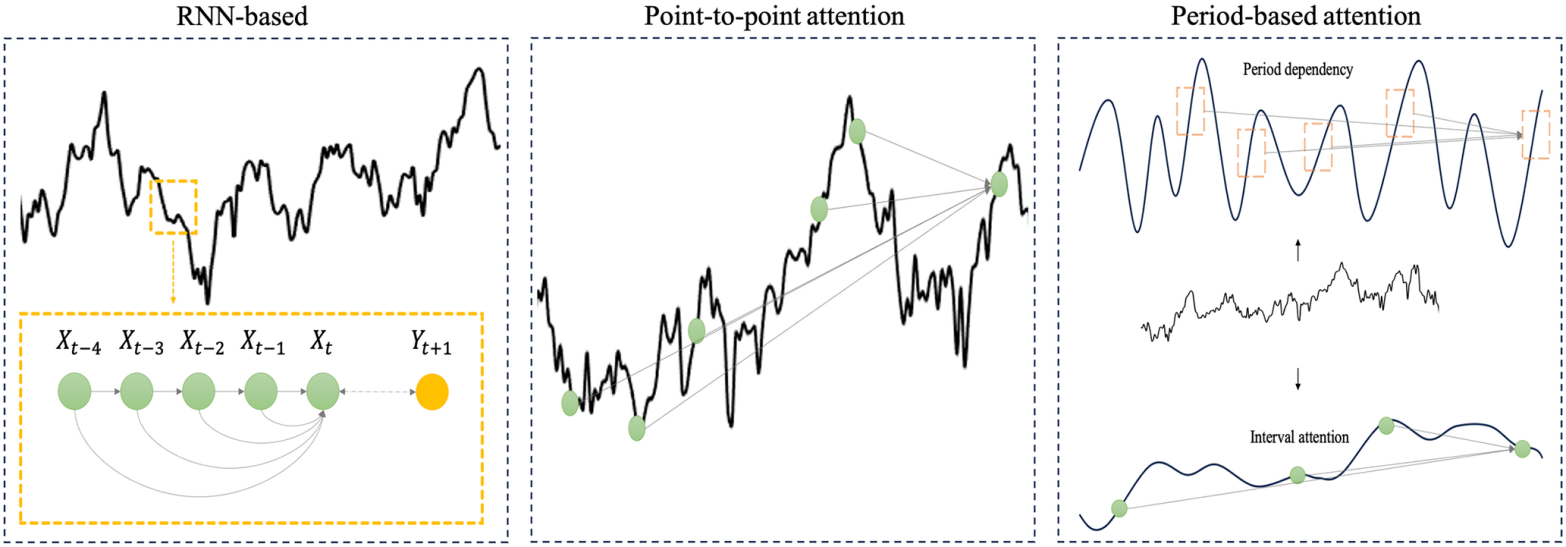
The different representation of financial time series. (Left) RNN-based models aggregate feature information within a time window to establish the mapping relationship with the next timestamp in that window. This approach falls short in utilizing data features beyond the specified window. (Middle) Transformer-based point-to-point attention mechanisms compute the impact of each historical time point on the current one, capturing global dependencies. Such high-granularity method is susceptible to noise, influencing attention weight calculations and potentially leading to model overfitting. (Right) Our approach decomposes sequence features into periodic fluctuations and trend directions. We use period-correlation to capture dependencies among periodic fluctuations, and the trend pattern is characterized by long-term smoothness. We employ a coarse-grained attention mechanism to capture dependencies.

Although these learning algorithms are aimed at handling noisy data and capturing complex, latent patterns, two main challenges hinder the solution to these problems: First, stock data are highly stochastic; as historical information is beneficial for prediction tasks, encoding stock features from a certain time window (e.g., 7 days, 30 days) is the most common method used in the representation stage that can capture dependencies such as a calendar pattern or an optimal roll window, as shown in Fig 1. Compared with textual data, stock data have a lower signal-to-noise ratio and more unobservable patterns. To address this issue, techniques from the field of natural language processing (NLP) can be directly applied to the stock market, overlooking the inherent characteristics of stock data. Second, previous studies have focused on point-to-point or period-to-point mechanisms such as Transformer and LSTM to predict stock movement. Although these methods can capture certain dependencies, they are limited in using this information for future market trends and their generalization capabilities are insufficient.

To address the barriers mentioned above, we develop an adaptive period-aggregation model named Latent Period-aggregated Stock Transformer (LPAST), which combines a variational autoencoder (VAE) with a period-to-period attention mechanism for multistep prediction in the stock market. Essentially, we view stock sequences as an aggregation of multiple patterns forming a whole, with the aim of identifying time series based on their latent representations that reflect the intricate characteristics of firms. In this process, the aim of the series decomposition from the perspective of frequency-domain analysis is to reduce noise interference and reveal latent patterns in the stock market. Additionally, we incorporate a self-correlation learning method into the model to guide the extraction of effective period patterns for the multistep prediction task. To aggregate complex multiple periods, we introduce routers to distribute the messages of different temporal indicators. The contributions of this study are as follows:

- We construct a novel period-aggregation representation scheme, in which the latent period correlation is accurately captured. To align with the characteristics of financial markets and reduce interference due to noise, a seasonal series decomposition module is developed to guide the next aggregation steps.
- We introduce a novel attention mechanism that integrates period information to automatically select period intervals and to adapt to long-term dependencies.
- We perform long-horizon prediction experiments to validate our proposed model and demonstrate its superiority over baseline models, including RNN-, Transformer-, and VAE-based models.

## Related works

The information required for predicting stock prices can be hidden within stock features. However, identifying the complex nonlinear relationships among these features is a challenging task in the stock market. In this section, we first provide a review of the relevant literature concerning conventional stock market models. Second, we describe the methods that capture short- and long-term dependencies in the stock market, forming the foundation for constructing our temporal aggregation framework.

### Seasonality effects in financial market

The seasonality effect refers to the cyclical and regular patterns in financial asset prices associated with specific calendar periods, such as certain months, trading days, intraday trading sessions, and particular holidays. Time series seasonality effects typically represent the overall market characteristics and trends from a market-level perspective. Depending on the time nodes of interest in research, these effects generally include the monthly effect, day-of-the-week effect, and holiday effect [8].

The “month effect” typically manifests as significantly different average returns in certain months of the year compared to others. The “January Effect” is one of the earliest documented month effects, observed by scholars [9]. It is characterized by average returns in January being significantly higher than those in other months. The “January Effect” is not confined to the U.S. stock market. Research by Gultekin (1983) [10] on stock markets in nearly 20 countries worldwide found that the “January Effect” is a prevalent phenomenon in these markets. The SAD effect (Seasonal Affective Disorder) is another significant month effect. Seasonal Affective Disorder is a well-documented medical condition characterized by mood disturbances resulting from changes in the body’s biological clock due to reduced daylight hours in the autumn and winter seasons. The SAD effect was first proposed by Kamstra (2003) [11] and refers to the abnormally low average stock returns during the shorter daylight periods of autumn and winter. The Day of the Week Effect, similar to the monthly effect, refers to the phenomenon where average returns on certain trading days within a week differ significantly from those on other days. Based on S&P 500 index return data from 1953 to 1970, Cross (1973) [12] found that the average return on Mondays was the lowest of the week, while the average return on Fridays was the highest.

Understanding seasonality effects is essential for advancing financial research. The seasonal effect, viewed as a temporal pattern, can be employed to comprehend and predict fluctuations in the financial markets. Consequently, many scholars apply statistical models to elucidate these fluctuations and identify future trends in financial time series.

### Statistical analysis of financial time series

Subjective models are employed in statistical methods, relying on empirical predictions based on past data. The prominent statistical learning methods include autoregressive moving average (ARMA) [13], autoregressive integrated moving average (ARIMA) [2], autoregressive conditional heteroscedasticity (ARCH) [14], and generalized autoregressive conditional heteroscedasticity (GARCH) [4]. Their frequent use across various domains is attributed to their simplicity and low complexity. These methods have successfully uncovered numerous financial phenomena to explain market fluctuations, referred to as ‘factors’, such as the Fama–French three–factor model [1] and the momentum factor [15]. However, an increasing number of factors that influence the stock market have been identified as research has progressed, leading to the creation of a ‘factor zoo’ [16]. Traditional statistical models are constrained in their ability to process the nonlinear dependencies inherent in high-dimensional factors.

Given the high dimensionality and dynamic nature of real financial markets, the applicability of single independent variable analysis methods is limited. In traditional financial research, principal component analysis (PCA) is commonly used to handle high-dimensional data. Lettau (2020) [17] combined principal component analysis with arbitrage pricing, extending the application of PCA to explain the co-movement of no-arbitrage factors in data. Introducing a no-arbitrage penalty term in PCA overcomes the issue of the low signal-to-noise ratio in financial data and yields information pertinent to kernel pricing. Additionally, Kelly (2020) [18] applied PCA to stock prediction models, employing directional PCA to guide unobservable dynamic factors through observable characteristics, thus obtaining a return/compensation relationship corresponding to risk compensation.

However, these methods often make assumptions that may not hold in real-world scenarios. The stock market is a complex system with numerous variables and interacting uncertainties, such as noise, policies, and manipulation.

### Deep learning in finance

Deep learning exhibits a superior capability to solve complex problems in many research areas including medicine [19], agriculture [20] and energy [21]. Fundamentally, deep learning is based on multilayered neural network structures, enabling incremental learning and the refinement of complex data representations [22].

Deep learning can transform various financial problems into challenges of learning relevant financial data representations particularly in complex financial settings involving numerous factor interactions [23, 24]. In the field of temporal modeling, Qin (2017) [25] introduced a dual-stage attention-based recurrent neural network to capture the long-term temporal dependencies in stock forecasting. Zhang (2017) [26] developed a variant of LSTM that decomposes the hidden states of memory units into multiple frequency components to capture trading patterns.

In recent years, with the increasing availability of financial data, scholars have begun to use unsupervised models to extract the features from stock market data. Given the vast volume of high-frequency financial data and their lack of labeling, Hou (2022) [27] introduced a contrastive multigrain learning framework (CMLF), which includes two innovative contrastive learning mechanisms and a gating mechanism for adaptive data fusion. This method was evaluated on three real stock markets and produced substantially improved results compared with those of the current leading systems, proving the effectiveness of multigranularity in stock trend prediction. Wang (2021) [28] introduced a contrastive predictive coding (Co-CPC) method based on joint distributions, aimed at reducing uncertainty through higher-accuracy stock representation from macro-level industry and micro-level hierarchical coupling, thereby addressing the weak generalization issues experienced in stock trend prediction. Co-CPC initially models the dependencies between a particular stock industry and the related macroeconomic variables, then learns stock representations through a self-supervised approach, which can be applied to downstream tasks such as stock trend prediction.

These methods, employing both supervised and unsupervised learning models, have yielded notable stock price predictions. However, these methods struggle to capture the long-term dependencies in financial time series due to the fixed window size inherent in RNN-based models.

### Transformers

Vaswani (2017) [29] proposed the Transformer model, which achieved success in natural language processing (NLP) tasks. The Transformer model is a sequence-to-sequence model that uses attention mechanisms to capture the (long-term) dependencies between input and output sequences, which has been developed in various fields such as computer vision, speech recognition, and video processing [30–32].

In the field of time series, Zhou (2021) [33] proposed an improved Transformer model specifically for long-term forecasting. Wu (2021) [34] designed a novel Transformer model incorporating an autocorrelation mechanism and a decomposition architecture, tailored for complex time series analysis. This indicates that Transformers achieve better performance in capturing long-term dependencies in time series prediction.

Transformers have successfully predicted stock movements, particularly when dealing with complex nonlinear relationships. Ding (2020) [23] introduced a multiscale Transformer framework that fuses the different structures of financial time series (intra-day and intra-week features). Wang (2022) [35] developed a reformed self-attention mechanism to identify the temporal pattern interactions in financial time series, and they constructed an unsupervised graph learning framework to reveal the implicit similarities among various stocks. Wang (2023) [36] proposed a multimodal and multitemporal tensor representation scheme that leverages stock correlations and different sources of market information based on the attention mechanism. Although these methods emphasize point-wise attention, computing attention for each timestamp, the temporal dependency in the stock market is complex and variable.

Unlike most existing models that use point-wise attention, which struggle with handling complex financial time series, we developed a period-aggregated attention mechanism. This proposed approach is guided by unsupervised series decomposition, adapted to the characteristics of the market.

## The proposed framework

The stock market is a complex and dynamic system filled with noisy information. Early studies typically incorporated historical information using either a fixed rolling time window (RNN-based model) or a point-to-point attention mechanism (Transformer). However, these methods do not adequately capture various latent temporal patterns and generalizations, leading to the loss of continuous information.

Given the *N* stocks list $S=\{s_{1},s_{2},s_{3},\ldots ,s_{N}\}$, at any trading day *t*, each stock *i* have an input sequence $X_{i}=\left\{x_{t-L},\ldots ,x_{t-1}\right\}\in R^{L\times F}$, where *L* is selected input length and *F* is the feature dimension. Our proposed model **F**(*X*_[1:*N*]_) aims to long-horizon prediction of daily return ratio, according to long-term time window(more than 2 months) of input sequence which would contain more period patterns.

**Fig 2:**
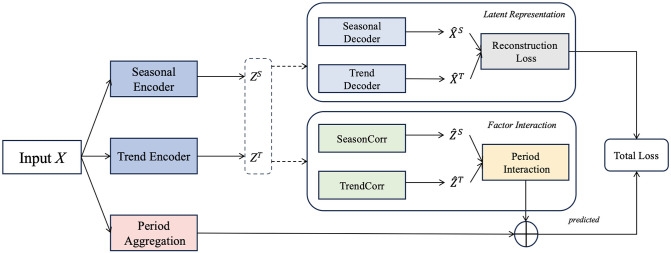
The proposed framework.

**Fig 3:**
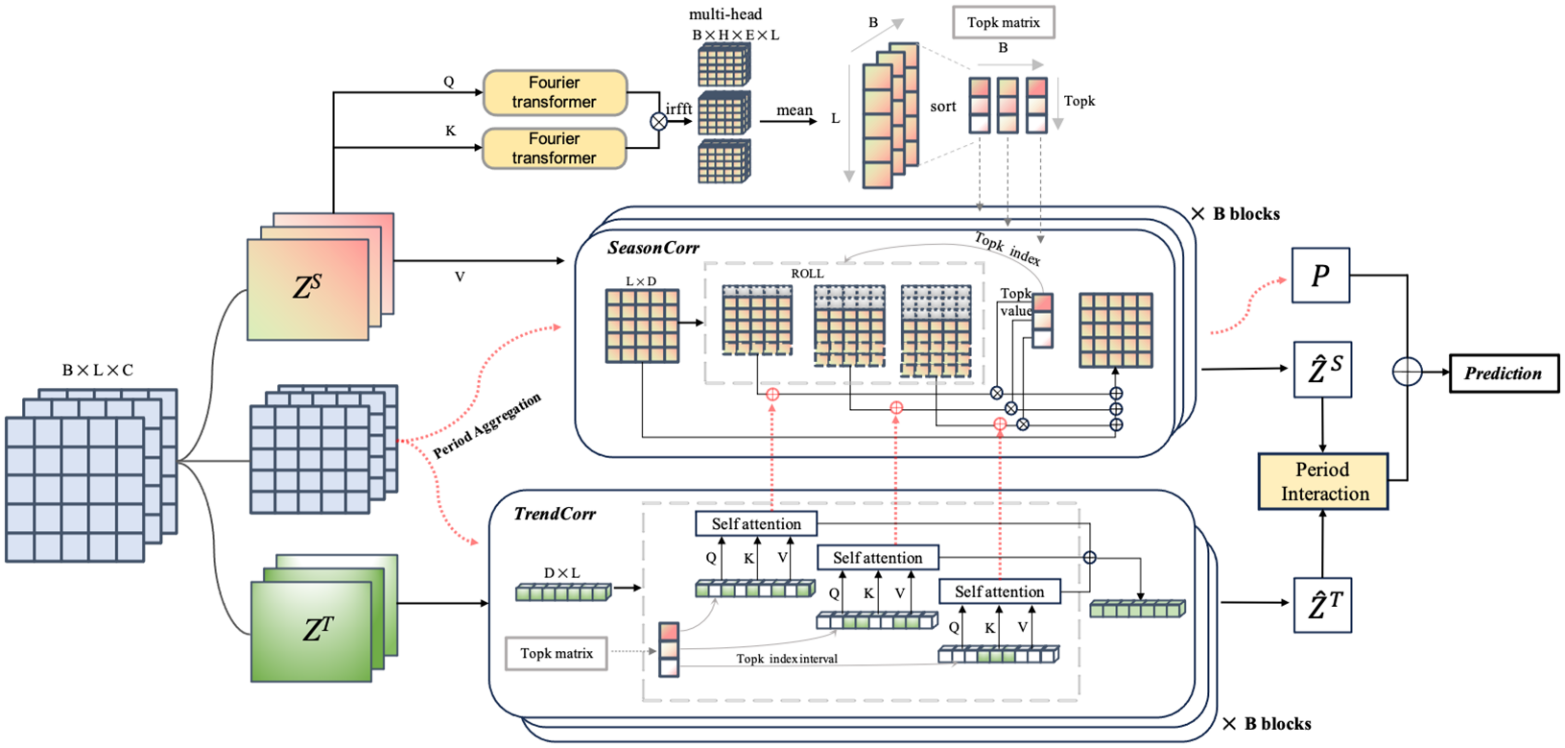
The details of period aggregation.

Fig 2 illustrates framework of our proposed approach. It consists of three major modules:**(i)Latent representation module** based on a Variational Autoencoder (VAE) that decomposes the input *X* into *Z*^*S*^ and *Z*^*T*^ using seasonal and trend encoders, respectively. These are then reconstructed into ${\hat{X}}^{S}$ and ${\hat{X}}^{T}$. This stage reduces noise and makes the embeddings better suited to stock market periods. **(ii)Period-based Transformer** with different attention mechanisms of *Z*^*S*^ and *Z*^*T*^(SeasonCorr and TrendCorr in Fig 2) to exploit intrinsic period patterns. We introduce more details of attention computation in Fig 3. **(iii)Period aggregation**(more details in Fig 3) encode input *X* by the above attention mechanisms, and then aggregate these two parts. **Period interaction** with a message passing mechanism by router to entangle ${\hat{Z}}^{S}$ and ${\hat{Z}}^{T}$.

### Representation learning of stock features

The effective representation of data plays a crucial role in deep learning, where different types of data require different representation methods. Fluctuations in the stock market exhibit periodic changes. Thus, learning the flexible representations of stock features is beneficial in forecasting tasks. Hirshleifer (2018) [37] offered evidence supporting the seasonality in the stock market. This trend is a noticeable shift in the time series of the stock market. However, processing representations of financial data is difficult without prior domain knowledge. Inspired by variational inference and information theory [38, 39], we used autocorrelation and mutual information (MI) to learn the latent representations and disentanglement mechanisms in a data-driven manner.

#### Variational autoencoder

Variational autoencoder (VAE) is an unsupervised generative learning model that learns the latent representations of the input data as random variables, which learns distribution of the latent features *z* given the input *x* and distribution of the input *x* given the latent features *z*. Similar to the conventional autoencoder, the VAE has an encoding process that converts the input into latent representations and a decoding process that reconstructs the original input using the learned representations. The distribution learning mechanism can be more useful when data is noisy, Duan [40] and Gu [41] demonstrated the robust performance of autoencoder-based methods in the stock market.

VAE learns the generative model as *p*(*x*, *z*) = *p*(*x*|*z*)*p*(*z*), where *x* is the input data and *z* is latent representation. *p*(*z*) is defined as a multivariate Gaussian distribution. VAE approximates the posterior with q(z|x)=N(z|μ(x),σ(x)), where mean and variance are determined by *x*. Then, VAE defines the learning problem as the maximum likelihood estimation of log *p*(*x*). Since directly computing this marginal likelihood is usually infeasible, we approximate it using variational inference and introduce the approximate posterior distribution *q*_*ϕ*_(*z*|*x*), the function can be formulated as:

$$\begin{array}{cc} \text{log}\;p_{\theta }\left(x\right) & =\text{log}\;\int p_{\theta }\left(x,z\right)\;dz\\ & =\text{log}\;\int q_{\phi }\left(z|x\right)\frac{p_{\theta }\left(x,z\right)}{q_{\phi }\left(z|x\right)}\;dz\\ & =\text{log}\;\int (\frac{p_{\theta }\left(x|z\right)p_{\theta }\left(z\right)}{p_{\theta }\left(z|x\right)}\frac{q_{\phi }\left(z|x\right)}{q_{\phi }\left(z|x\right)})\;dz\\ & =\text{log}\;\int p_{\theta }\left(x|z\right)\;dz+\text{log}\;\int \frac{q_{\phi }\left(z|x\right)}{p_{\theta }\left(z|x\right)}\;dz-\text{log}\;\int \frac{q_{\phi }\left(z|x\right)}{p_{\theta }\left(z\right)}\;dz\\ & \underset{ELBO}{\underset{︸}{\geq \text{log}\;\int p_{\theta }\left(x|z\right)\;dz-\text{log}\;\int \frac{q_{\phi }\left(z|x\right)}{p_{\theta }\left(z\right)}\;dz}} \end{array}$$ (1)

where *θ* is parameters for each linear layers of encoder, and *ϕ* is parameters for each linear layers of decoders. Applying Jensen’s inequality to the logarithm of the yields the Evidence Lower Bound(ELBO), which consists of two main components: the expected log-likelihood term and the Kullback-Leibler(KL) divergence term. It can be transformed as:

$$\begin{array}{c} L_{ELBO}=E_{z∼q_{\phi }\left(x|z\right)}\left[\text{log}\;p_{\theta }\left(x|z\right)\right]-KL(q_{\phi }\left(z|x\right)||p\left(z\right)) \end{array}$$ (2)

Given the conditions of *x*, *q*_*ϕ*_(*z*|*x*) is the distribution of *z* inferred with *ϕ* i.e., the specific data of *z* inferred with *x* as input and *ϕ* as a parameter. *p*_*θ*_(*x*|*z*) is the distribution of *x* inferred with *θ*. And KL is the Kullback-Leibler divergence between *q*_*ϕ*_(*z*|*x*) and *p*(*z*), Specially, the KL divergence of two distributions *q*(*x*) and *p*(*x*) measures their similarity and is defined as:

$$\begin{array}{c} KL\left(q\left(x\right)\|p\left(x\right)\right)=\sum_{x}q\left(x\right)\frac{q(x)}{p(x)}=E_{q(x)}\frac{q(x)}{p(x)} \end{array}$$ (3)

The first term in Eq (2) maximizes the conditional probability of *x* given the latent representation *z*. It can be seen as the reconstruction loss. The second term minimizes the difference between the prior and the approximated posterior.

#### Latent seasonal-trend representation

The variational autoencoder (VAE) effectively captures the intrinsic features in data through deconstruction and reconstruction, independent of human experience. Stock market data constitute intricate time series featuring various temporal patterns, including seasonality and trends. Targeted adjustments to the model are essential to effectively capture these phenomena.

Consider sequences denoted as $X_{1:T}^{\left(i\right)}=x_{1}^{\left(i\right)},x_{2}^{\left(i\right)},\ldots ,x_{t}^{\left(i\right)},\ldots ,x_{T}^{\left(i\right)}$, where *i* ∈ 1, 2, …, *N*, and each $x_{t}\in R^{F}$ is an input vector at time step *t*, where *F* is the feature dimension. Specially, we encode all stock series subsequences *X* as seasonal part *Z*^*S*^ and trend part *Z*^*T*^.

$$\begin{array}{c} Z^{S}=Q_{\phi^{S}}\left(Z^{S}|X\right)=N\left(\mu^{S}\left(X\right),\sigma^{S}\left(X\right)\right) \end{array}$$ (4)

$$\begin{array}{c} Z^{T}=Q_{\phi^{T}}\left(Z^{T}|X\right)=N\left(\mu^{T}\left(X\right),\sigma^{T}\left(X\right)\right) \end{array}$$ (5)

The encodings *Q*(*Z*^*S*^|*X*) and *Q*(*Z*^*T*^|*X*) represent the distributions of the seasonal and trend parts, respectively, given the input time series *X*, assuming Gaussian distributions. According to Eqs (2) and (3) of Evidence Lower Bound(ELBO) in VAE, the loss function of our VAE-based module can be formulated as:

$$\begin{array}{c} L_{VAE}=\underset{reconstruction\;\;loss}{\underset{︸}{E_{{Q_{\phi }}^{S}\left(Z^{S}|X\right)}\left[logP_{\theta^{S}}\left(X^{S}|Z^{S}\right)\right]+E_{{Q_{\phi }}^{T}\left(Z^{T}|X\right)}\left[logP_{\theta^{T}}\left(X^{T}|Z^{T}\right)\right]}}\\ \underset{KL\;\;divergence}{\underset{︸}{-KL(Q_{\phi^{S}}\left(Z^{S}|X\right)\|P\left(Z^{S}\right))-KL(Q_{\phi^{T}}\left(Z^{T}|X\right)\|P\left(Z^{T}\right))}} \end{array}$$ (6)

However, directly measuring the encodings is impossible due to the unknown variables *X*^*S*^ and *X*^*T*^. Combining these two terms leads to confusion as the decoder may struggle to accurately reconstruct the complex time series from each representation. The loss function of VAE $L_{VAE}$ can be decomposed into two parts: the reconstruction loss and the KL divergence. We can estimate the reconstruction loss using the following formula, with the assumption of a Gaussian distribution:

$$\begin{array}{c} L_{r}=-\sum_{\tau =1}^{T-1}\|R_{XX}\left(\tau \right)-R_{{\hat{X}}^{S}{\hat{X}}^{S}}{\left(\tau \right)\|}^{2}+CORT\left(X,{\hat{X}}^{T}\right)-\|\hat{X^{T}}+{\hat{X}}^{S}-X{\|}^{2} \end{array}$$ (7)

$$\begin{array}{c} CORT\left(X,{\hat{X}}^{T}\right)=\frac{\sum_{i=1}^{t-1}\Delta X_{i}^{T}{\hat{X}}_{i}^{T}}{\sqrt{\sum_{i=1}^{t-1}\Delta X^{T}}\sqrt{\sum_{i=1}^{t-1}\Delta \hat{X^{T}}}} \end{array}$$ (8)

where $L_{r}$ is the reconstruction loss part of $L_{VAE}$. $\hat{X}$ is the reconstruction of *X*, decoded by *Z*. $R_{XX}\left(\tau \right)=\underset{L\to \infty }{\text{lim}}\frac{1}{L}\sum_{t=1}^{L}X_{t}X_{t-\tau }$ is the autocorrelation function of *X*_*t*_ and *τ* is the lag. $R_{XX}\left(\tau \right)$ reflects the time-delay similarity between *X*_*t*_ and its lag series *X*_*t*−*τ*_. The autocorrelation is a measure of the similarity between a given time series and a lagged version of itself over successive time intervals. $CORT(X,{\hat{X}}^{T})$ reflects the temporal correlation and Δ*X*_*i*_ = *X*_*i*_ − *X*_*i*−1_ is the first difference.

We introduce additional mutual information regularization terms to the loss function to alleviate the divergence narrowing problem of the KL term. Through this regularization, model can increase the mutual information between *Z*^*S*^, *Z*^*T*^ and *X*, as well as decrease the mutual information between *Z*^*S*^ and *Z*^*T*^. The total loss of the representation learning stage can be composed of: **(i)Reconstruction loss. (ii)KL divergence. (iii)Mutual information regularization**.

The formula can be expressed as:

$$\begin{array}{c} L_{VAE}=L_{r}-L_{KL}+I\left(X,Z^{S}\right)+I\left(X,Z^{T}\right)-I\left(Z^{S},Z^{T}\right)\\ L_{KL}=KL(Q_{\phi^{S}}\left(Z^{S}|X\right)\|P\left(Z^{S}\right))+KL(Q_{\phi^{T}}\left(Z^{T}|X\right)\|P\left(Z^{T}\right)) \end{array}$$ (9)

where $L_{r}$ is the reconstruction loss, $L_{KL}$ is the KL divergence and *I*(⋅, ⋅) denotes the mutual information between two representations. Then $L_{VAE}$ will be incorporated into the proposed model’s loss calculation with a certain weight in the next section.

### Temporal patterns encoder

We introduce specific learning algorithms tailored for distinct temporal patterns into the representation learning process. Autocorrelation effectively captures the periodic patterns in stock market data through identifying similarities in lagged periods due to the regularity in the cyclical fluctuations of the stock market. Taking inspiration from Transformers, we integrate a period-to-period attention mechanism using autocorrelation into the proposed model. The Transformer model is a sequence-to-sequence model that uses attention mechanisms to capture the dependencies between input and output sequences. Most Transformer models consider the features of all nodes when calculating attention weights. However, the traditional Transformer model ignores the local dependencies among the nodes in financial time series. Here, we take the representation sequences *Z*^*S*^ and *Z*^*T*^ as the initial input, according to Eqs (4) and (5).

Fig 3 illustrates period aggregation. First, we apply the Fourier transform to process *Q*^*S*^ and *K*^*S*^, computing their autocorrelation and converting it into an attention map called the Topk matrix. The Topk matrix identifies the top *k* elements (including index and value) based on autocorrelation similarity for each stock. For example, in the stock market, the most common periods are daily, weekly, and monthly patterns, represented as [1, 7, 30] in the Topk(*k* = 3) matrix. This method allows us to discover more relevant and latent temporal patterns compared to fixed patterns. According to the Topk matrix, $\hat{Z^{S}}$ and $\hat{Z^{T}}$ are generated by the *SeasonCorr*(*Z*^*S*^) and *TrendCorr*(*Z*^*T*^) respectively which would be processed in period interaction stage. According to the Topk matrix, $\hat{Z^{S}}$ and $\hat{Z^{T}}$ are generated by *SeasonCorr*(*Z*^*S*^) and *TrendCorr*(*Z*^*T*^), respectively, and then processed in the period interaction stage. The hidden outputs of *SeasonCorr*(*X*) and *TrendCorr*(*X*) generate *E*, which is added to the output of the period interaction for the final prediction.

#### Period-matterd aggregation

The input $Z^{j}\in R^{B\times L\times D}$ (*j* includes the seasonal or trend part) can be transformed into query *Q*^*j*^, key *K*^*j*^ and value *V*^*j*^ after the projector, where *L* is the length of the sequence and *D* is the dimension of the representation. In the period-matter aggregation, we firstly use the Fourier transform to calculate the autocorrelation and select *k* most periodic elements(indexes and values).

$$\begin{array}{c} \text{FF}=F^{-1}\left(F\left(Q^{S}\right)\cdot \bar{F(K^{S})}\right)\;\;\;,\text{FF}\in R^{B\times H\times E\times L}\\ \text{Matrix}=sort{\left(\frac{1}{H\times E}(\sum_{h=1}^{H}\sum_{e=1}^{E}{\text{FF}}_{b,h,e,l})\right)}_{\left[:,:k\right]}\;\;\;,\text{Matrix}\in R^{B\times k} \end{array}$$ (10)

We set *τ*_1_ ⋯ *τ*_*k*_ as the index of $\text{Matrix}\in R^{B\times k}$ which represent timestamps included in each period. For example, *τ* = 3 represents this is a 3-days period. The value of **Matrix** can be denoted as *υ*(*τ*_1_) ⋯ *υ*(*τ*_*k*_), the weight of values can be generated by *SoftMax* function. It can be expressed as:

$$\begin{array}{c} \hat{\upsilon }\left(\tau_{1}\right),\cdots ,\hat{\upsilon }\left(\tau_{k}\right)=SoftMax\left(\upsilon \left(\tau_{1}\right)\cdots \upsilon \left(\tau_{k}\right)\right) \end{array}$$ (11)

$$\begin{array}{c} {\hat{Z}}^{S}\left(Q^{S},K^{S},V^{S}\right)=\sum_{t=1}^{k}Roll\left(V^{S},\tau_{t}\right)\hat{\upsilon }\left(\tau_{t}\right) \end{array}$$ (12)

where *k* = |*a* × log *L*|, *a* is hyperparameter; *Roll*(*V*, *τ*) denotes the right shift of the sequence *V* by *τ*.

The temporal patterns in seasonal subseries exhibit a consistent phase position across periods. Thus, the period-based dependencies link the subseries within estimated periods. The trend represents the long-term change direction in a time series and illustrates the overall trend in the data over time. Here, interval aggregation is used to capture the long-term dependencies in the trend subseries. We apply the *τ*_1_ ⋯ *τ*_*k*_ as the interval timestamps to generate *Z*^*T*^(*τ*_1_) ⋯ *Z*^*T*^(*τ*_*k*_). For example, the trend representation *Q*^*T*^(*τ* = 1) = [*q*_1_, **q**_**1**_, *q*_3_, **q**_**3**_, *q*_5_, **q**_**5**_, ⋯] if *Q*^*T*^ = [*q*_1_, *q*_2_, *q*_3_, *q*_4_, *q*_5_, *q*_6_ ⋯]. According to the index of **Matrix**, the ${\hat{Z}}^{T}$ can be denoted as:

$$\begin{array}{c} Q^{T}\left(\tau_{t}\right),K^{T}\left(\tau_{t}\right),V^{T}\left(\tau_{t}\right)=Interval_{\tau_{t}}\left(Q^{T},K^{T},V^{T}\right) \end{array}$$ (13)

$$\begin{array}{c} {\hat{Z}}^{T}\left(Q^{T},K^{T},V^{T}\right)=\frac{1}{k}\sum_{t=1}^{k}Attention\left(Q^{T}\left(\tau_{t}\right),K^{T}\left(\tau_{t}\right),V^{T}\left(\tau_{t}\right)\right) \end{array}$$ (14)

${\hat{Z}}^{S}$ and ${\hat{Z}}^{T}$ are generated to capture the short-term and long-term temporal dependencies based on the latent period patterns. Subsequently, we combine the autocorrelation map and attention map, which are produced during the generation of ${\hat{Z}}^{S}$ and ${\hat{Z}}^{T}$ respectively. The dimension of $\hat{\upsilon }\left(\tau_{t}\right)$ is *B*, when $\text{Matrix}\in R^{B\times k}$ we expand and repeat it to obtain a tensor **U**(*τ*_*t*_) of dimension *B* × *H* × *E* × *L*.

$$\begin{array}{c} PeriodAgg\left(X\right)=\sum_{t=1}^{k}Roll\left(V_{X}^{S},\tau_{t}\right)\left(U\left(\tau_{t}\right)+Softmax(\frac{Q_{X}^{T}\left(\tau_{t}\right)K_{X}^{T}\left(\tau_{t}\right)}{\sqrt{d}})\right) \end{array}$$ (15)

where $d=\frac{E}{H}$ and $V_{X}^{S},Q_{X}^{T},K_{X}^{T}$ is generated during the *SeasonCorr*(*X*) and *TrendCorr*(*X*) as shown in Fig 3 (red lines).

#### Period interaction

Self-supervised decomposition and period-based attention can be effectively used to capture seasonal-trend patterns. In the real stock market, these patterns mutually influence each other, forming an intricate and complex system. Additionally, the interactions among various factors influence stock asset prices. For these interactions, we introduce the interaction stage after period aggregation, which can capture the cross-period dependencies among seasonal-trend series.

**Fig 4:**
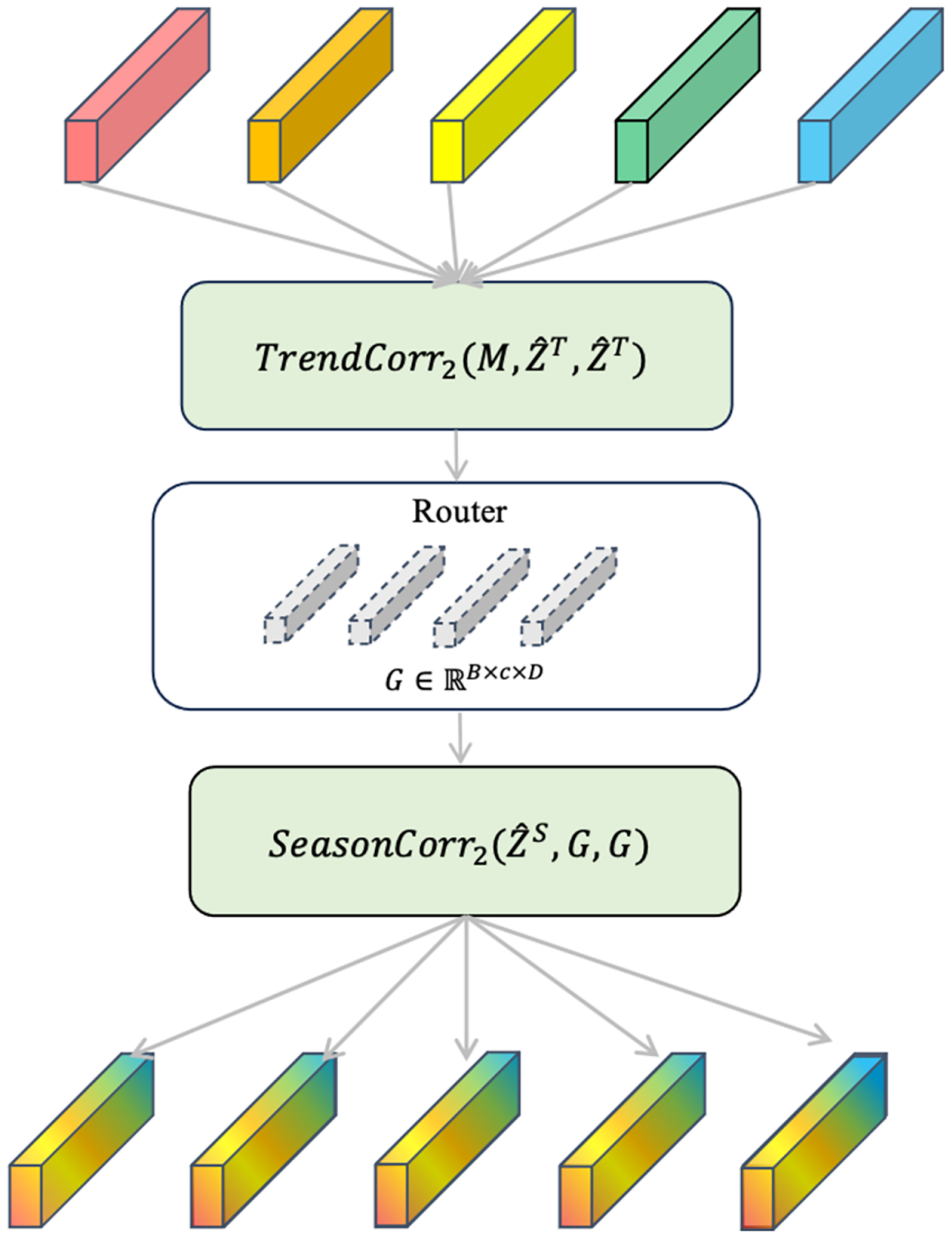
The illustration of period interaction.

As shown in Fig 4, we propose a routing mechanism to pass information from hidden features of ${\hat{Z}}^{S}$ and $\hat{Z^{T}}$ with a learnable matrix. We first use routers as query in *TrendCorr* and all vectors of $\hat{Z^{T}}$ as key and value to aggregate messages from trend patterns. Then *SeasonCorr* receive the information by using aggregated messages as key and value and $\hat{Z^{S}}$ as query. The input from period aggregation stage can be expressed as:

$$\begin{array}{c} \begin{array}{cc} & \hat{Z^{S}}=SeasonCorr_{1}\left(Z^{S},Z^{S},Z^{S}\right)\\ & \hat{Z^{T}}=TrendCorr_{1}\left(Z^{T},Z^{T},Z^{T}\right)\\ & P=PeriodAgg_{1}\left(X,X,X\right) \end{array} \end{array}$$ (16)

where the fully connected layer transforms the representation *Z* and raw input *X* into *Q*, *K*, and *V* in the attention layer; $\hat{Z^{S}}$ and $\hat{Z^{T}}$ capture the local dependency from seasonal and trend series, respectively; and *P* aggregates the global temporal information.

Then, we apply new *SeasonCorr* and *TrendCorr* with routers, the routing mechanism can be built in:

$$\begin{array}{c} G=TrendCorr_{2}\left(M,\hat{Z^{T}},\hat{Z^{T}}\right)\\ \hat{G}=SeasonCorr_{2}\left(\hat{Z^{S}},G,G\right)\\ \hat{P}=LayerNorm\left(P+\hat{G}\right)\\ {\text{Out}}_{final}=LayerNorm\left(\hat{P}+MLP\left(\hat{P}\right)\right) \end{array}$$ (17)

where $G\in R^{B\times c\times D}$ is the aggregated messages from all dimensions; $M_{i}\in R^{B\times c\times D}$ is the learnable routing vector as the router; *c* is a fixed number to integrate temporal information; ${\hat{G}}_{i}\in R^{B\times L\times D}$ captures the cross-period interaction.

#### Objective function

We employed a combined strategy during the training process to effectively balance representation ability and fitting capability. The predicted stock return ratio ${\hat{r}}_{i,t}$ for stock *i* at time *t* is obtained using a dense layer to the **Out**_*final*_, which is generated by Eq (17). We then calculate the MSE and ranking losses using weighting parameter λ. The MSE loss aims to minimize the difference between the prediction and ground truth, whereas the ranking loss aims to preserve the relative order to the maximum extent possible.

$$\begin{array}{c} L_{final}=\sum_{i=1}^{N}\|{\hat{r}}_{i,t}-{\hat{r}}_{j,t}{\|}^{2}+\lambda \sum_{i=1}^{N}\sum_{j=1}^{N}max\left(0,-\left({\hat{r}}_{i,t}-{\hat{r}}_{j,t}\right)\cdot \left(r_{i,t}-r_{j,t}\right)\right) \end{array}$$ (18)

We define a consolidated end-to-end loss function, represented by the weighting coefficient *α*, integrating the supervisory ranking loss with the self-supervised representation proximity loss $L_{VAE}$ from Eq (9). The total loss of proposed model can be formulated as:

$$\begin{array}{c} L=L_{final}+\alpha L_{VAE} \end{array}$$ (19)

## Results

### Experiment setup

We conduct experiments to test the effectiveness of the proposed latent representation and period aggregation framework for predicting long-term movements of financial time series. We initially assess the performance of the proposed model after detailing the implementation of the experiments by comparing the model with other prediction methods. Subsequently, we discuss an ablation study involving the proposed module. Finally, we conduct a simulation to evaluate the proposed model.

#### Data collection

In the experiments section, we validate the effectiveness of our framework using real-world financial time series including stock data, options data and exchange data as shown in Table 1. **(i)Stock data**: to evaluate the efficacy of the proposed framework in predicting stock movements, we conducted a series of experiments using real market data from S&P 500, NASDAQ and CSI 300 firms spanning from February 10, 2020, to November 18, 2023. Each piece of data includes eight features: Volume, Turnover, Change, Change rate, Highest price. The daily transaction data were sourced from Wharton Research Data Services (WRDS) and China Stock Market & Accounting Research Database (CSMAR). **(ii)Option data** [42]: The SSE 50 ETF is the first stock index option in China, and we gathered daily price of option contracts from February 9, 2015, to October 9, 2021. Since the proposed model aims to identify multiple periodic patterns and make long-term predictions, we chose option contracts with expiration dates exceeding 150 days. **(iii)Exchange data** [34, 43]: The Exchange data is a collection of daily exchange rates of eight different countries from 1990 to 2016.

**Table 1:** Statistics of datasets.

Datasets	Length	Dimension	Num	Loss function	Evaluation
S&P 500	932	5	198	L in Eq (19)	MSE+MRR
NASDAQ	932	5	83	L in Eq (19)	MSE+MRR
CSI 300	954	5	230	L in Eq (19)	MSE+MRR
ETF-Option	155	1	452	MSE	MSE
Exchange-Rate	7,588	1	8	MSE	MSE

#### Evaluation metrics

Time series prediction involves regression and optimal selection problem. Hence, we use the Mean Square Error (MSE), Mean Reciprocal Rank (MRR) and Cumulative Investment Return Ratio (IRR) to evaluate our proposed model’s performance of stock data. These metrics that have been widely adopted in prior studies [35, 44, 45]. The formulas for the MSE and MRR are as follows:

$$\begin{array}{c} MSE=\frac{1}{n}\sum_{i=1}^{n}{\left(y_{i}-{\hat{y}}_{i}\right)}^{2} \end{array}$$ (20)

$$\begin{array}{c} MRR=\frac{1}{L}\sum^{L}\frac{1}{Q}\sum_{i=1}^{Q}\frac{1}{{\text{rank}}_{i}} \end{array}$$ (21)

Here, *L* represents the prediction length; *Q* denotes the number of query stocks; and *rank*_*i*_ is the predicted ranking position of stock *i*. A lower MSE value and a higher MRR value are indicative of superior performance. Besides, the ETF-Option and Exchange data are univariate time series that we only apply MSE to train and evaluate.

#### Experiment setting

**Fig 5:**
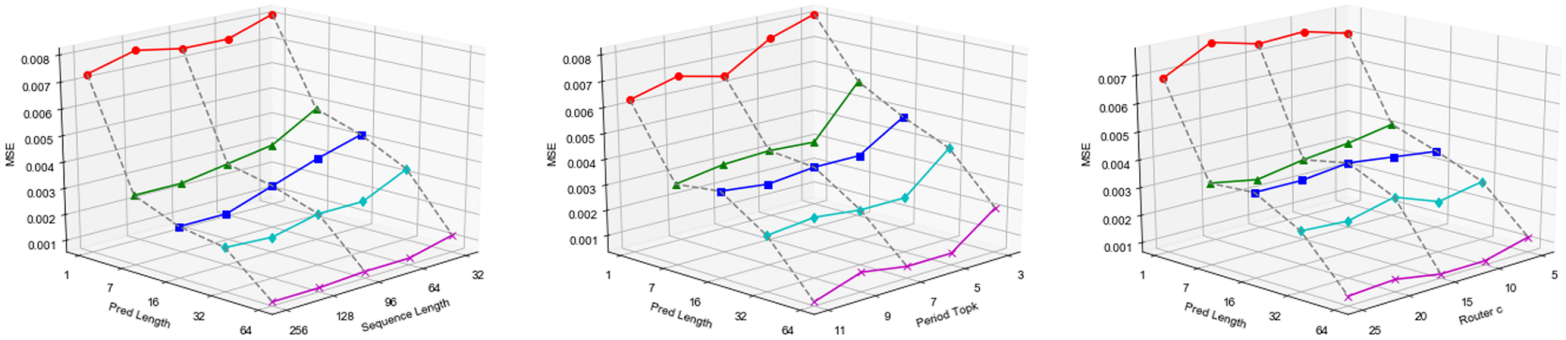
MSE values for different input length *L*, period selection *k* and router *c*.

**Fig 6:**
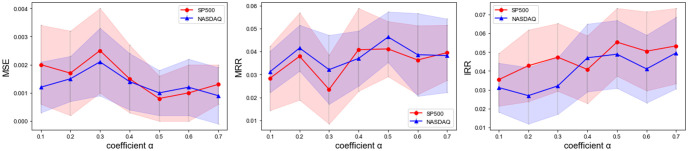
Varying coefficient *α* of unsupervised fusion loss.

We employ the standard normalization for all stock data sets. Our method is trained with combined loss according to Eq (19), using the ADAM optimizer with an initial learning rate of 10^−4^. We apply the grid search to select the optimal hyperparameters regarding MSE. The top-k periods selection *k* (in Eq (11)) is searched within {3, 4, 5, …, 11}, and the message distribution router *c* (in Eq (17)) is searched within {5, 10, 15, 20, 25}. From Fig 5, the prediction performance is best when *k* = 5, *c* = 10. The input length is searched within {32, 64, 96, 128, 256} for Stock and Exchange data, while {32, 48, 64} for Option data due to its total length. In this study, we set *L* = 128 for Stock and Exchange data and *L* = 64 for Option data. We tune unsupervised and rank loss weight *α* and λ within {0.1, 0.2, …, 0.7} according to Feng(2019) [44], and set loss hyperparameters to *α* = 0.5 and λ = 0.5 as illustrated in Fig 6, and the result shows that a lower standard error and improved performance are achieved when *α* fall within the range of 0.4 to 0.6. The dimension of representation features *D* is searched within {32, 64, 128, 144, 256} and set to *D* = 64.

The training process is stopped within 100 epochs. We divide the transaction days into three periods. The first 70% days is used for training, the following 20% days is used for validation, and the last 10% days are applied for testing. Our evaluation follows a rolling window approach, as recommended by Li (2020) [46]. Specifically, we use historical information from the previous *L* transaction days to forecast stock movements over a prediction length *O* of 7, 16, 32, and 64 transaction days.

We train the model 10 times using different initialization for each method compared in our experiments to increase the robustness of our evaluation. We rank these 20 runs, selecting the top five based on their performance during the validation period, report the average performance of these selected runs in the testing phase, to mitigate the effect of fluctuations due to random initialization.

### Experimental results

#### Baselines

We compare our models with other dynamic temporal prediction models:

- FactorVAE [40] is a probabilistic dynamic temporal stock prediction model based on VAE.
- D-Va [47] combines hierarchical VAE and the diffusion probabilistic approach for multistep stock prediction.
- REL [48] is a self-supervised model based on contrastive learning that captures the latent relation attribution of stocks.
- HMG-TF [23] applies Gaussian Transformer to daily and weekly trading series.
- Transformer [29] is the basic Transformer model.
- Adv-ALSTM [49] simulates the stock dynamics with adversarial training and LSTM.

#### Comparison

Table 2 shows the predictions of all baseline models and our model. We set prediction length *O* = {7, 16, 32, 64}, the larger the length, the harder the making prediction. The best result is highlighted in bold face in this table. Specifically, under the setting of *L* = 128 and *O* = 64 in Stock datasets, LPAST achieves **16.7%** (0.0012→0.0010) reduction of MSE in NASDAQ, **77.1%** (0.0035→0.0008) in S&P 500 and **19.8%** (0.1023→0.0820) in CSI 300. And it yields **18%** averaged improvement of MRR, while **25%** (NASDAQ), **29%** (S&P 500) and **10%** (CSI 300) of IRR. For univariate time series, LPAST gives at least 19% (0.3046→0.2459) reduction of MSE in Exchange data. And it provides 13% (*O* = 32) and 8% (*O* = 64) MSE reduction, but does not achieve better performance in Option data under the settings of *L* = 64, *O* = {7, 16}.

**Table 2:** Performance comparison with different prediction length *O* ∈ {7, 16, 32, 64}.

Model		LPAST			FactorVAE			D-Va			REL			HMG-TF			Transformer			Adv-ALSTM		
		MSE	MRR	IRR	MSE	MRR	IRR	MSE	MRR	IRR	MSE	MRR	IRR	MSE	MRR	IRR	MSE	MRR	IRR	MSE	MRR	IRR
NASDAQ	7	**0.0021**	**0.0485**	0.039	0.0038	0.0413	**0.048**	0.0041	0.0321	0.021	0.0044	0.0357	0.015	0.0083	0.0311	0.014	0.0033	0.0463	0.033	0.0054	0.0258	0.038
	16	**0.0034**	**0.0421**	**0.031**	0.0058	0.0405	0.021	0.0053	0.0287	0.028	0.0075	0.0293	0.030	0.0151	0.0268	0.021	0.0051	0.0368	0.029	0.0098	0.0229	0.031
	32	**0.0013**	**0.0383**	**0.062**	0.0079	0.0366	0.025	0.0032	0.0252	0.018	0.0090	0.0245	0.028	0.0059	0.0285	0.033	0.0068	0.0329	0.021	0.0854	0.0136	0.045
	64	**0.0010**	**0.0463**	**0.048**	0.0092	0.0381	0.033	0.0012	0.0276	0.011	0.0065	0.0286	0.012	0.0081	0.0167	0.036	0.0045	0.0316	0.022	0.0720	0.0142	0.009
S&P 500	7	**0.0038**	**0.0513**	0.018	0.0046	0.0425	**0.039**	0.0054	0.0354	0.025	0.0041	0.0404	0.023	0.0065	0.0301	0.021	0.0093	0.0421	0.038	0.0045	0.0302	0.015
	16	**0.0037**	**0.0683**	**0.063**	0.0059	0.0468	0.031	0.0067	0.0219	0.022	0.0092	0.0378	0.022	0.0098	0.0232	0.019	0.0053	0.0518	0.031	0.0083	0.0208	0.032
	32	**0.0025**	**0.0446**	0.028	0.0050	0.0410	**0.041**	0.0063	0.0208	0.014	0.0052	0.0276	0.038	0.0073	0.0079	0.013	0.0077	0.0365	0.022	0.0092	0.0115	0.025
	64	**0.0008**	**0.0411**	**0.055**	0.0035	0.0329	0.017	0.0072	0.0215	0.033	0.0087	0.0159	0.026	0.0072	0.0368	0.014	0.0097	0.0363	0.013	0.0061	0.0119	0.039
CSI 300	7	0.0664	0.0393	0.023	**0.0479**	**0.0509**	0.031	0.0951	0.0377	0.029	0.0652	0.0239	0.023	0.0930	0.0350	0.020	0.0811	0.0356	**0.032**	0.1041	0.0306	0.018
	16	0.0998	**0.0586**	**0.059**	0.0783	0.0512	0.029	0.1359	0.0381	0.031	0.0850	0.0230	0.029	**0.0618**	0.0353	0.015	0.0620	0.0321	0.043	0.2387	0.0335	0.041
	32	**0.0523**	**0.0519**	**0.065**	0.1892	0.0498	0.054	0.1142	0.0315	0.019	0.0809	0.0345	0.028	0.1069	0.0331	0.021	0.1580	0.0420	0.039	0.2954	0.0238	0.023
	64	**0.0820**	**0.0544**	**0.051**	0.1023	0.0436	0.046	0.2022	0.0408	0.018	0.1375	0.0317	0.023	0.1291	0.0299	0.036	0.1955	0.0227	0.028	0.2820	0.0152	0.019
Univariate series																						
Option	7	0.1861	-	-	0.3205	-	-	0.3339	-	-	0.2033	-	-	0.1647	-	-	**0.1525**	-	-	0.2628	-	-
	16	**0**.2839	-	-	0.4144	-	-	0.2621	-	-	0.2801	-	-	**0.2419**	-	-	0.3772	-	-	0.3259	-	-
	32	**0.3317**	-	-	0.3839	-	-	0.3766	-	-	0.4556	-	-	0.4050	-	-	0.4339	-	-	0.6244	-	-
	64	**0.3895**	-	-	0.5973	-	-	0.5748	-	-	0.4790	-	-	0.4258	-	-	0.5364	-	-	0.8698	-	-
Exchange	7	**0.2459**	-	-	0.3821	-	-	0.5134	-	-	0.6456	-	-	0.3989	-	-	0.3551	-	-	0.3046	-	-
	16	**0.2896**	-	-	0.4845	-	-	0.8589	-	-	0.6934	-	-	0.3278	-	-	0.5623	-	-	0.8987	-	-
	32	**0.2922**	-	-	0.9688	-	-	1.3023	-	-	1.1367	-	-	0.3712	-	-	0.5056	-	-	1.4390	-	-
	64	**0.3893**	-	-	1.1902	-	-	1.2289	-	-	0.9923	-	-	0.4978	-	-	0.4012	-	-	1.4656	-	-

Overall, Adv-ALSTM is an RNN-based model that primarily considers the short-term dependency of stock series and overlooks the broader temporal influence. Transformer and HMG-TF performed better in Option data, because they can capture global temporal dependencies through point-to-point attention at each timestamp. However, this point-wise attention is susceptible to noise data, particularly in the stock market.

FactorVAE, D-Va, and REL are VAE-based models that extract the latent representation of stock series using a self-supervised approach, emphasizing the unique patterns inherent in the stock. LPAST is also the VAE-based model combining the advantages of Transformers by applying a period-to-period attention. It outperforms the baseline models in long-term prediction, especially in complex datasets. Compared to RNN-based and Transformer-based models, LPAST maintains better robustness when input information becomes richer and noisier.

### Ablation study

Our framework consists of two dominant modules: the period-base attention, the decomposition and fusion of latent representation part. In the section, we present the results of ablation experiments to analyze these elements.

#### Effectiveness of period aggregation

**Fig 7:**
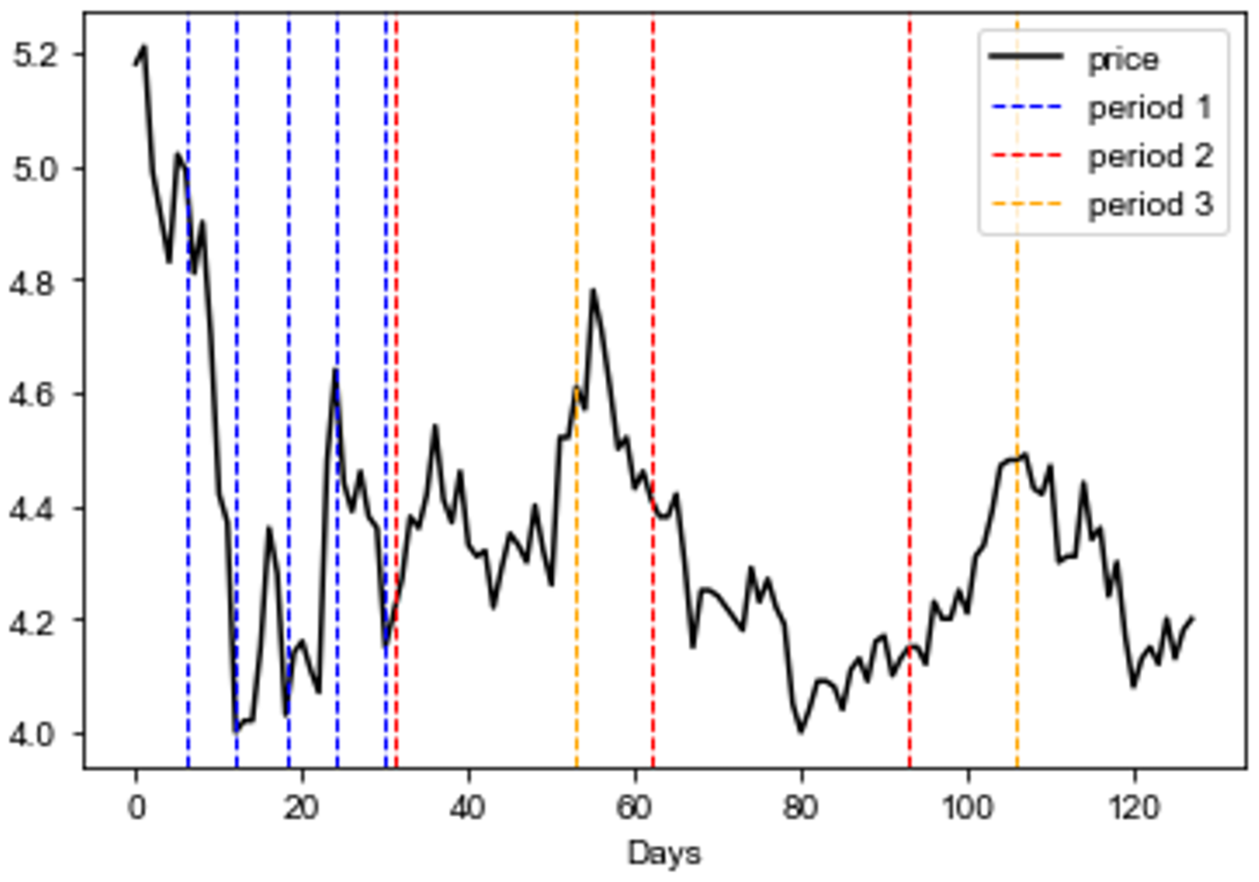
Visualization of period aggregation. we set topk to 3 which means model would capture three latent periods. Period 1 had a shorter interval, representing weekly or daily dependency; Periods 2 and 3 represented longer period dependencies, such as monthly or quarterly.

In finance, the influence of temporal dependency, such as lag, is well acknowledged. A common strategy in pilot work is the use of an RNN-based model to capture local dependencies or Transformer-based models to capture global dependencies, which are both point-wise interactions. We think that the period-wise interaction of the latent vector is more important for stock prediction because of the seasonality of stock series. We used a different attention computation, replacing the period-wise attention in Eqs (16) and (17), to judge weather incorporation with period-wise interaction could further boost the performance of our model. We find that period-wise attention is superior to point-wise attention, which achieved the best performance, as shown in Table 3. This finding shows that the period-to-period attention mechanism in our proposed model effectively captures period patterns, which can be help with investment decisions. Fig 7 depicts an example of a latent period dependency resulting from the period aggregation mechanism. The proposed method can capture more unobservable periods and is more generalizable compared with the fixed temporal window of the RNN-based model and the point-to-point attention mechanism.

**Table 3:** Ablation of attention mechanism.

Market Pred-length		S&P 500			
		7	16	32	64
PeriodAggregation	MSE	**0.0038**	**0.0037**	**0.0025**	**0.0008**
	MRR	**0.0513**	**0.0683**	**0.0446**	**0.0411**
	IRR	**0.018**	**0.063**	**0.028**	**0.055**
FullAttention	MSE	0.0052	0.0128	0.0093	0.0835
	MRR	0.0425	0.0323	0.0236	0.0351
	IRR	0.015	0.017	0.018	0.021
LogSparseAttention	MSE	0.0041	0.0073	0.0084	0.0307
	MRR	0.0507	0.0376	0.0439	0.0362
	IRR	0.018	0.018	0.027	0.035
LSHAttention	MSE	0.0045	0.0072	0.0128	0.0295
	MRR	0.0490	0.0370	0.0435	0.0365
	IRR	0.018	0.020	0.028	0.025

#### Effectiveness of latent representation

We introduce a latent representation module designed to extract the latent features of stock series. Contrary to earlier methods that directly embed stock features without any regularization, our module leverages an unsupervised methodology. We conduce a series of experiments to analyze the performance of the representation part, which we compare with two primary methods in the stock prediction task and one decomposition method in time-series analysis. As shown in Table 4, LSTM and CNN embeddings are commonly used for single-day prediction or binary classification; proposed approach, which combines supervised and unsupervised representations, exhibits better performance. For avgpool decomposition, the formula is as follows:

$$\begin{array}{c} Z^{T}=AvgPool\left(Padding\left(X\right)\right)\\ Z^{S}=X-Z^{T} \end{array}$$ (22)

**Table 4:** Ablation of representation mechanism.

Market Pred-length		S&P 500			
		7	16	32	64
LatentST(Ours)	MSE	**0.0038**	**0.0037**	**0.0025**	**0.0008**
	MRR	**0.0513**	**0.0683**	**0.0446**	**0.0411**
	IRR	**0.018**	**0.063**	**0.028**	**0.055**
Avgpool decomposition	MSE	0.0141	0.0273	0.0133	0.0122
	MRR	0.0292	0.0262	0.0219	0.0233
	IRR	0.007	0.018	0.017	0.023
LSTM embedding	MSE	0.0057	0.0102	0.0074	0.0078
	MRR	0.0398	0.0327	0.0383	0.0301
	IRR	0.015	0.016	0.015	0.021
CNN embedding	MSE	0.0093	0.0078	0.0069	0.0065
	MRR	0.0409	0.0349	0.0395	0.0318
	IRR	0.016	0.017	0.016	0.025

This is a linear decomposition method commonly used for time series with a high signal-to-noise ratio, but its performance may be limited in the stock market.

#### Investment simulation

**Fig 8:**
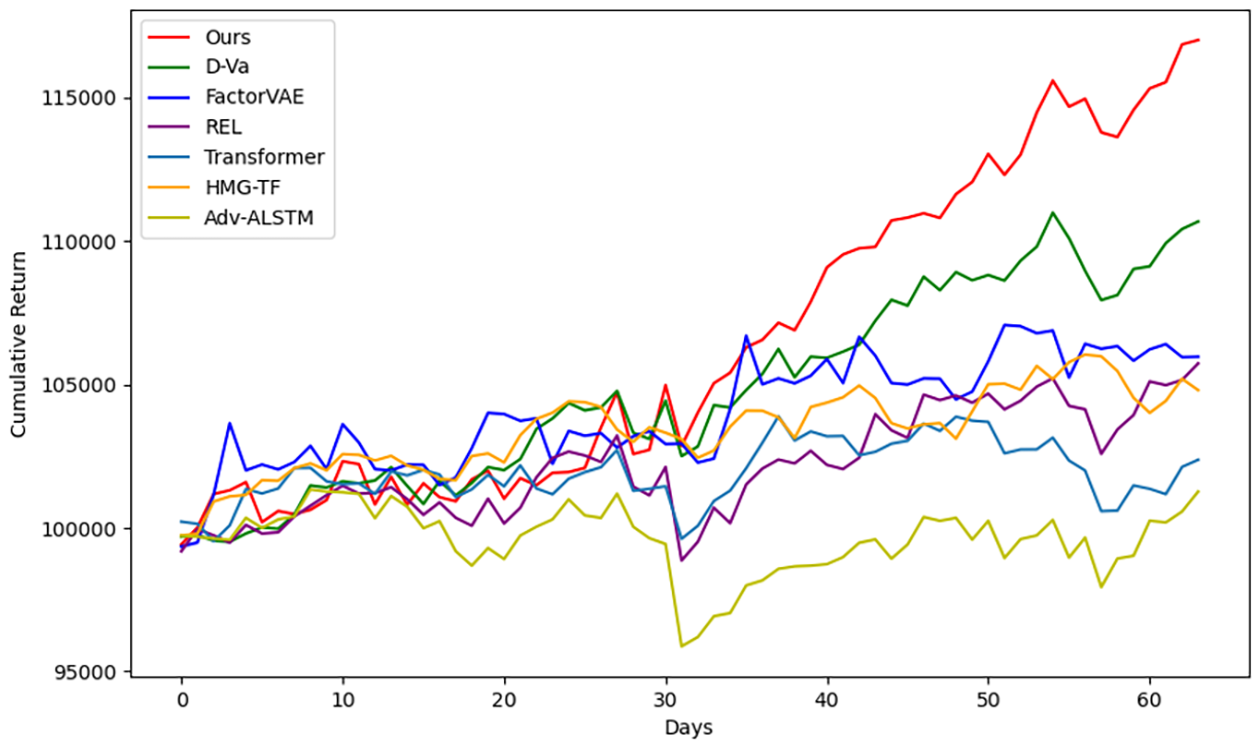
Investment simulation (no transaction fees).

We conduct a stock investment simulation using 60 days of SP500 data from February to April 2018 to further evaluate our model. We compare our method with the above-mentioned six baseline methods based on initial capital of USD 100,000, with the investment strategy being to buy and sell the top five stocks with the highest return ratio on the current day. We always maximize stock purchases by selecting stocks from the top five list with equal share allocations. Fig 8 illustrates the performance of these models over 60 days. Our model (red line) performs the best (USD 117,221) at the end of the period. The FactorVAE model (blue line) performs better during the initial period, and the Transformer-based model achieve similar performance toward the end of the period. This indicates that the VAE-based models had stronger generalization ability, and our aggregation method yields the highest return in long-term predictions.

## Discussion

Our work has achieved an innovative integration by seamlessly combining unsupervised learning with supervised learning. Specifically, by leveraging Variational Autoencoder (VAE), we effectively analyze the seasonal and trend characteristics of sequences, offering robust guidance for subsequent forecasting tasks. Additionally, in the next process, the period-based Transformer fully exploits the features extracted by the VAE. Based on the seasonal effects examined in traditional financial research, we utilize VAE to extract seasonal characteristics from the data. Unlike prior studies [40, 41], we introduce the mutual information penalty term into the loss function to simultaneously learn and reconstruct both seasonal and trend representations. Furthermore, from frequency domain analysis perspective, we propose a period-to-period attention mechanism. In contrast to traditional RNN models, our approach considers the diversity of potential periodic features, rather than relying on fixed time windows based on experience. Additionally, we have refined the attention mechanism to mitigate noise susceptibility arising from dependencies on relationships across global time points.

In high-dimensional stock markets, the proposed model has demonstrated robust and outstanding results in long-term forecasting tasks. As shown in Table 2, the model exhibits strong predictive performance across three stock markets, performing particularly well in the U.S. stock market compared to the Chinese stock market. From the perspective of the Efficient Market Hypothesis, research indicates that the Chinese stock market is less efficient than the U.S. stock market, resulting in stock information not fully reflecting its intrinsic values [50, 51]. This also highlights proposed model’s capability to capture the intrinsic value of stocks. Additionally, the T+1 trading system and the 10% daily price fluctuation limitation in the Chinese stock market may contribute to the model’s insufficient short-term predictive capability (prediction length *O* = {7, 16}). In univariate time series, due to lower market complexity (dimension *D* = 1), the predictive performance of our model shows only a slight improvement over benchmark models. Moreover, in experiments with Option data (input length *L* = 64), the short-term predictive ability of model is inferior to Transformer-based models. This could be due to lower noise and shorter sequence length in low-dimensional markets, where point-to-point attention can more effectively capture global time dependencies. These observations also underscore the limitations of our model, when data length or dimensional information is insufficient, the model’s performance may be somewhat constrained.

Traditional time series models in financial research often struggle to handle high-dimensional information, while models from computer science are typically not designed to address the specific characteristics of financial markets. This study leverages deep learning for high-dimensional modeling and feature extraction to demonstrate the existence and effectiveness of latent periodic features in financial markets. Consequently, we conclude that reasonable quantification of periodic features can more effectively capture market fluctuations. This integration of computer technology and financial research promotes the capability of modeling to represent complex financial markets. Fig 8 illustrates the model’s excellent performance in investment simulations, demonstrating that this research has practical implications for portfolio management in real markets. In future research, we plan to incorporate additional financial market data, including stock market data from various countries and high-frequency trading data, to further explore the integration of efficient market theory and deep learning. Additionally, we aim to investigate how to combine the model with other financial theories and methods to develop a more comprehensive and holistic financial intelligence platform.

## Conclusion

Deep learning models have achieved significant success in financial time series forecasting by capturing nonlinear relationships that traditional quantitative models often fail to identify. This study employs deep learning models to represent the seasonal effects in financial research, addressing the limitations of current RNNs and Transformers in capturing latent periodic features. Experiments were conducted on stock, option and exchange data, with results demonstrating the superior performance of proposed model in long-horizon prediction task. Additionally, we performed ablation studies and visualization analyses, further confirming the robustness of the model. A detailed analysis of the experimental results was also conducted, exploring the practical implications of this research and discussing directions for future studies.

## Supporting information

S1 Data (ZIP)
